# Template-assisted formation of microsized nanocrystalline CeO_2_ tubes and their catalytic performance in the carboxylation of methanol

**DOI:** 10.3762/bjnano.2.86

**Published:** 2011-11-30

**Authors:** Jörg J Schneider, Meike Naumann, Christian Schäfer, Armin Brandner, Heiko J Hofmann, Peter Claus

**Affiliations:** 1 Department of Chemistry, Eduard-Zintl-Institute, Inorganic Chemistry, Technische Universität Darmstadt, Petersenstr. 18, 64287 Darmstadt, Germany; 2 Department of Chemistry, Ernst-Berl-Institute, Technical Chemistry II, Technische Universität Darmstadt, Petersenstr. 20, 64287 Darmstadt, Germany

**Keywords:** activation of CO_2_, ceria, electrospinning, exotemplating, nanotubes

## Abstract

Polymethylmethacrylate (PMMA)/ceria composite fibres were synthesized by using a sequential combination of polymer electrospinning, spray-coating with a sol, and a final calcination step to yield microstructured ceria tubes, which are composed of nanocrystalline ceria particles. The PMMA template is removed from the organic/inorganic hybrid material by radio frequency (rf) plasma etching followed by calcination of the ceramic green-body fibres. Microsized ceria (CeO_2_) tubes, with a diameter of ca. 0.75 µm, composed of nanocrystalline agglomerated ceria particles were thus obtained. The 1-D ceramic ceria material was characterized by X-ray diffraction, scanning electron microscopy (SEM), high-resolution transmission electron microscopy (HRTEM), UV–vis and photoluminescence spectroscopy (PL), as well as thermogravimetric analysis (TGA). Its catalytic performance was studied in the direct carboxylation of methanol with carbon dioxide leading to dimethyl carbonate [(CH_3_O)_2_CO, DMC], which is widely employed as a phosgene and dimethyl sulfate substitute, and as well as a fuel additive.

## Introduction

Ceria, CeO_2_, is known as a semiconducting ceramic material with unique electronic properties, exhibiting a broad range of functional properties with potential for application in various areas [1–3]. Due to its extraordinary thermal and chemical stability, it is a promising material for catalytic, environmental and energy applications, such as in solid oxide fuel cells (SOFCs) [4–5], or for the elimination of pollutants from automobile exhaust gases, or for fluid catalytic cracking or dehydrogenation of ethylbenzene to styrene. With respect to catalysis, CeO_2_ is a valuable support material for the low-temperature water–gas shift reaction and preferential oxidation of CO in hydrogen-enriched atmospheres. Herein, we will focus our attention on the direct carboxylation of methanol to dimethyl carbonate (DMC) catalyzed by nanostructured ceria. DMC is a noncorrosive and environmentally friendly solvent, which is used as alternative to highly toxic carbonylating and methylating agents. For catalytic applications, the performance of ceria is strongly dependent on its crystallinity and textural properties, including surface area and porosity. Although nanocrystalline ceria is known to be more active than amorphous ceria [6], it tends to agglomerate into larger crystallites under conditions of high-temperature catalysis. In this context, the preparation of high-surface-area ceria films by using a polymer-templating method was investigated in an effort to reduce such agglomeration [1,7–11]. Nanosized ceria can be synthesized by methods such as chemical vapour deposition (CVD), spray pyrolysis, hydrothermal synthesis or electrosynthesis [1–2,9]. These approaches lead to particulate, nanocrystalline powdered samples, with the exception of the CVD method, which gives thin ceria films [2–15]. One-dimensional (1-D) nanostructures such as nanowires, nanorods and nanotubes have attracted increasing attention owing to their reduced dimensionality and unique functional properties [16].

Electrospinning is a technology that allows the formation of polymer fibres with nanoscale dimensions [17–20]. Such nanofibres and nanotubes based on electrospun polymers offer a broad range of applications in areas such as photonics, sensorics, catalysis, medicine, pharmacy and functional textiles [17,20]. By employing these one-dimensional (1-D) polymer fibres as structure-directing templates for nanomaterial synthesis, 1-D oxide materials are accessible. This process is called TUFT (tubes by fibre templates) [17–18] and typically uses an electrospinning technique in which polymer and inorganic precursor solutions are electrosprayed *together* to give the final inorganic 1-D material.

By taking advantage of the higher catalytic activity of nanocrystalline ceria compared to amorphous ceria on one side [6], and the possibility to obtain stable 1-D microstructured oxide morphologies by electrospinning, we designed a material combining the advantages of a nano/micro-structured hierarchy, which offers a high catalytic activity on the nanoscale, combined with a low tendency for the isolated nanoparticles to further agglomerate when using a pre-assembly technique to form a microsized 1-D wire structure. We employed a template-directed synthesis using electrospun polymer fibres, followed by deposition of a nanoscaled inorganic ceria sol-precursor solution by spray coating onto the polymer fibre template. The sequential electrospinning and spraycoating process steps are finally followed by dry O_2_ plasma etching and calcination to yield microsized ceria tubes composed of nanocrystalline, entangled ceria tubes which display a macroscopic matlike material morphology. Our process, however, is significantly different to the TUFT process in which both components, i.e., the polymer and the inorganic precursor component, are sprayed at the same time. The macroscopically sized ceramic mats, which are composed of porous hierarchically structured microsized nanocrystalline ceria tubes, were thus obtained by a sequential synthesis process. As a suitable application, the catalytic performance of these macrosized mats of nanostructured ceria tubes was investigated for the direct carboxylation of methanol with carbon dioxide leading to dimethyl carbonate (DMC).

## Results and Discussion

### Synthesis of polymer fibre templates

**Figure 1:**
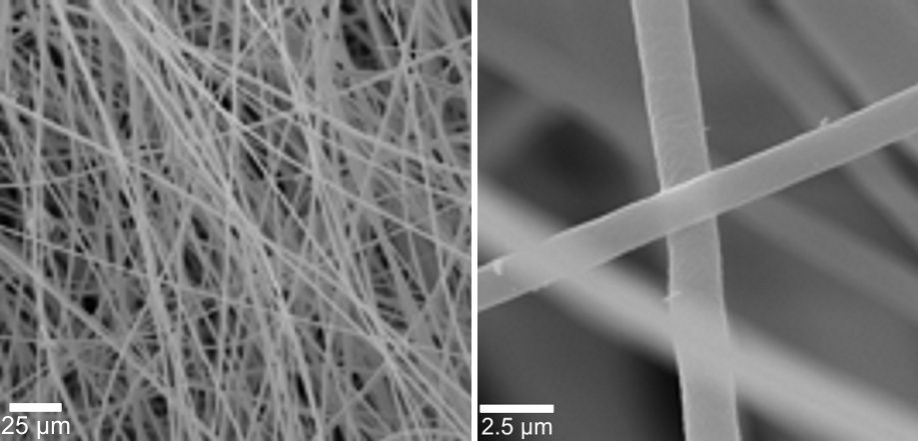
Scanning electron micrographs (SEM) of electrospun PMMA fibres, fabricated from a 15 wt % solution of PMMA in a mixture of acetone and dimethyl formamide (60/40). An average fibre diameter of 1.8 μm was obtained.

Polymer fibres were fabricated by electrospinning in a vertical electrode arrangement. The polymer solution was 15 wt % PMMA dissolved in a mixture of acetone and dimethyl formamide (60/40), which was electrospun at 26 kV through a nozzle with dimensions of 0.8 × 4.0 mm and a current flow of 4 μA was achieved. The electrospun polymer template has an average fibre diameter of 1.3–1.8 μm, which can be varied by changing the properties of the polymer solution used [17]. Typically, dense mats of fibres on the counter electrode (copper plate, 15 × 15 cm^2^) were formed (Figure 1). The thickness of the obtained fibre mats depends on the spinning time.

### Synthesis and characterization of hierarchical microsized nanocrystalline ceria fibre mats

#### Formation of ceria tubes without surfactant

**Scheme 1:**
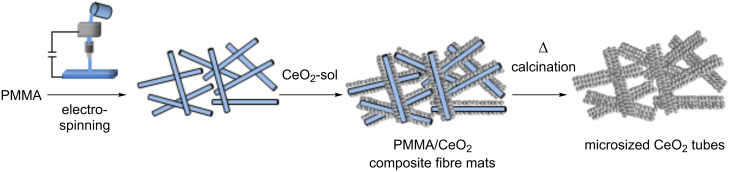
Schematic representation of the exotemplating process for the preparation of CeO_2_ fibre mats with a microtubular structure. After electrospinning of a PMMA solution, the resulting PMMA fibre mat is impregnated with the ceria sol suspension by spray coating and then precalcined giving a PMMA/CeO_2_ green-body composite. A final calcination step leads to the microsized pure ceria fibre mats composed of ceria tubes.

**Figure 2:**
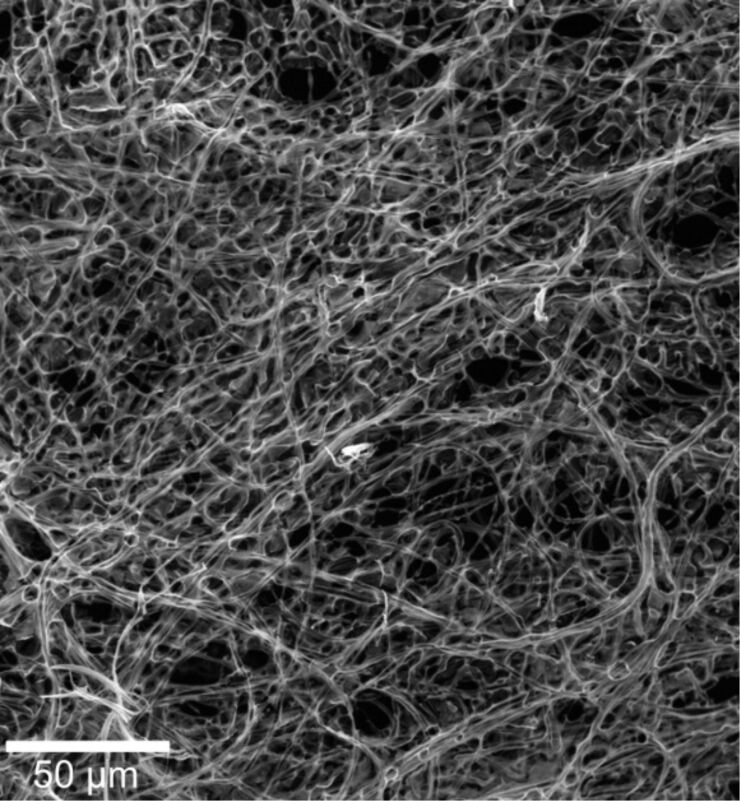
SEM image of the macrosized ceria mats, composed of ceria microtubes obtained by plasma treatment and further calcination at 350 °C for 3 h.

Ceria tubes were prepared by an exotemplating technique. After controlled ageing of a sol-precursor solution prepared from cerium ammonium nitrate (NH_4_)_2_Ce(NO_3_)_6_ in water and ammonia at 50 °C for half an hour, the sol was allowed to infiltrate into the electrospun polymer-template fibre mats upon application by a spray-coating technique [9,20–21]. The obtained PMMA/ceria composite samples were then plasma etched in 20 vol % oxygen atmosphere (air) for 16 h to remove the majority of the polymer template. Removal of the PMMA solely by a thermal process, through calcination of the polymer/inorganic hybrid structure, results in a complete collapse of the resulting porous ceria structure and formation of a dense ceria film. The plasma etching process was followed by a final calcination of the “green-body structure” at 350 °C for 3 h. Scheme 1 shows the complete synthesis process in an overview. Figure 2 shows a SEM image of the thus-obtained ceria mats composed of ceria tubes after the final ceramisation step.

**Figure 3:**
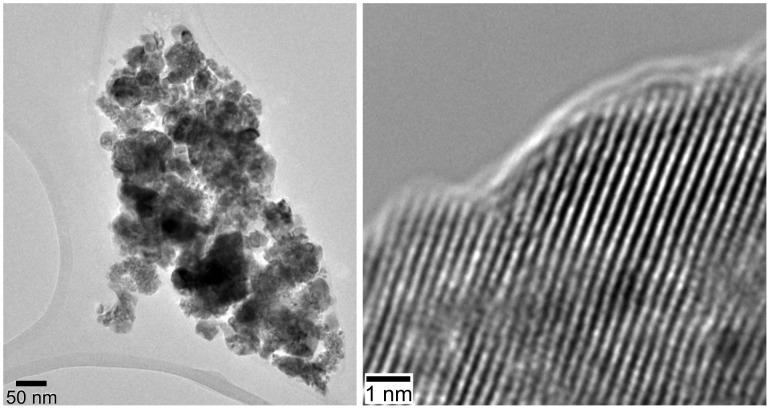
TEM and high-resolution TEM images of agglomerated nanosized ceria particles, which are the building blocks of the microscopic ceria tubes shown in Figure 2. These tubes are entangled into larger aggregates, which are the building blocks for the ceria mats. The particles are isolated from the dense mats by intense ultrasonification over a long period.

The nanocrystallinity of the ceria tubes was investigated by transmission electron microscopy (TEM, Figure 3). Samples were obtained by ultrasonification over a long period, which breaks down the microtubular structure, of which the ceria mats are composed, and results in spherical ceria particles, which are clustered into larger micrometre-sized aggregates.

**Figure 4:**
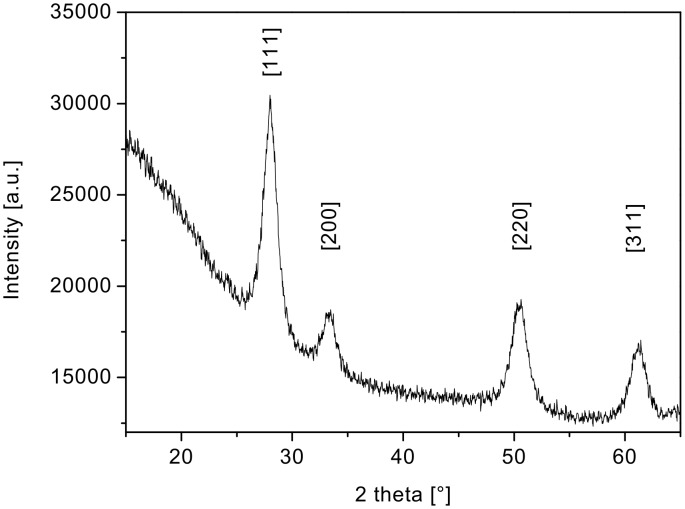
X-ray diffraction (XRD) pattern of nanostructured ceria.

Figure 4 shows the XRD spectrum of such ceria nanoparticles [22]. The spectrum indicates a phase-pure face-centred-cubic fluorite-type CeO_2_ (JCPDS 78-0694, No. 225). No other phases or impurities were detected.

#### Formation of ceria tubes with surfactant Pluronic P123^®^

**Scheme 2:**
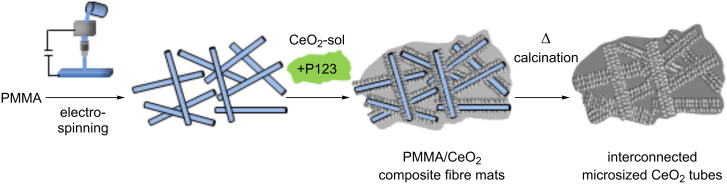
Schematic representation of the exotemplating process for the preparation of CeO_2_ fibremats by using triblock copolymer Pluronic P123^®^. After the formation of the PMMA fibremats by electrospinning, the ceria sol as well as P123^®^ were added simultaneously, dried and plasma etched, giving a 2-D ceria/1-D ceria interconnected structure of the green-body composite. A final calcination step yielded the ceria tube structure in which the individual tubes are interconnected by a ceria thin film (dark grey).

**Figure 5:**
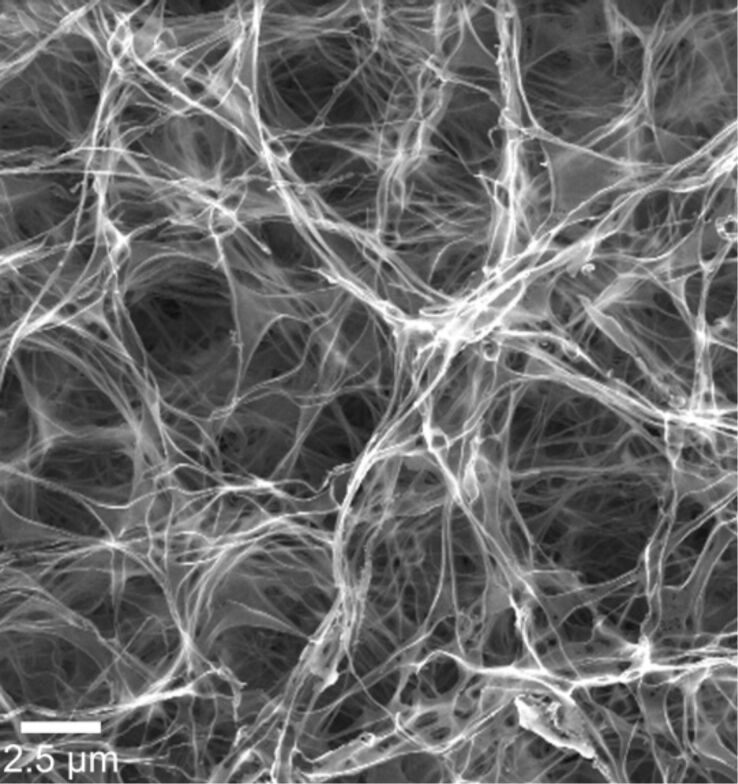
SEM image of the final ceria mats, obtained by plasma treatment and further calcination at 350 °C, for 3 h to remove the polymer template. The ceria-sol-impregnation step of the polymer fibres was performed with the addition of Pluronic P123^®^.

To further improve the intimate contact between the aqueous ceria sol (see before) and the polymer fibre substrate during synthesis, the surfactant Pluronic P123^®^ was added to the sol prior to spray coating. Pluronic P123^®^ is a triblock copolymer based on individual poly(ethylene glycol)-poly(propylene glycol)-poly(ethylene glycol) blocks, which form spherical and cylindrical micelles, and which could thus allow for a better contact of the inorganic ceria sol with the electrospun polymer fibres during impregnation. After spray-coating followed by sol–gel transformation to the ceramic green body at 80 °C overnight, the green body was further plasma etched (20 vol % O_2_ atmosphere for 16 h), followed by a calcination step at 350 °C for 3 h, yielding the final macrostructured ceria mats (Scheme 2 and Figure 5). Surface area measurements employing the Brunauer–Emmett–Teller (BET) method, revealed a surface area of 126 m^2^·g^−1^ for this hierarchically structured ceria material.

**Figure 6:**
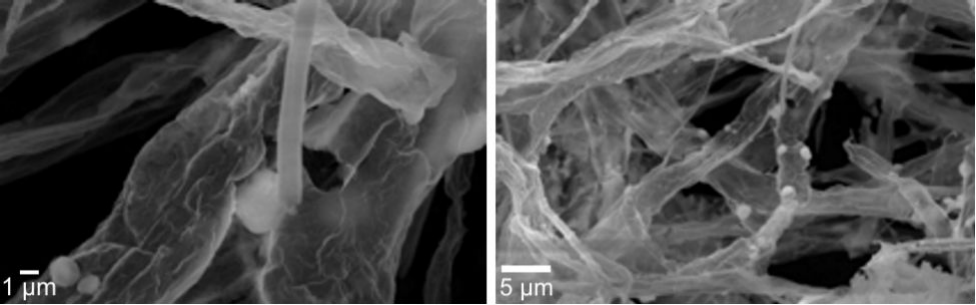
SEM images (different magnifications) of interconnected microsized ceria tubes. Samples were fabricated, using PMMA fibre templates, coated with a ceria sol containing surfactant Pluronic P123^®^.

After a final calcination step at 350 °C, the morphology of the ceria material changed considerably compared to the ceria material obtained by the previously described procedure without Pluronic P123^®^ additive. Again, ceria tubes of microscopic size were obtained with comparable diameter as before (diameter ca. 0.75 μm). However, these were instead embedded in a thin ceria film which interconnects the individual ceria tubes, building up a filamentous network structure (Figure 6).

**Figure 7:**
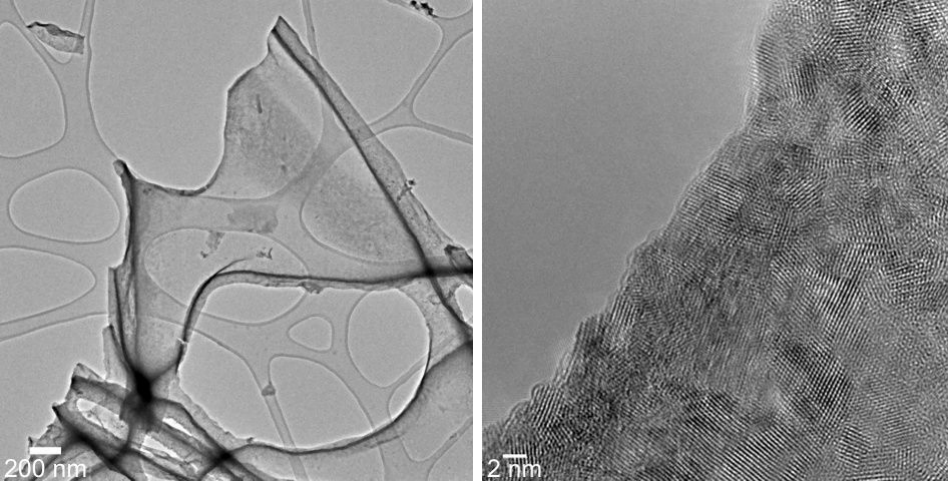
TEM (left) and HRTEM (right) images of nanostructured ceria thin film interconnecting the ceria tubes (with addition of Pluronic P123^®^ to the sol) at different magnifications.

The crystalline nature of this ceria thin film interconnecting the tubes is shown in the TEM images (Figure 7). Obviously the block copolymer P123^®^ is capable of acting as a template to guide the ceria sol around the polymer fibres, resulting in 1-D ceria tubes after polymer etching and calcinations, as found for the process without addition of the block copolymer. The 2-D ceria film formed due to the addition of the block copolymer P123^®^ interconnects these ceria tubes, thus forming a network structure. Although we were not able to determine the thickness of the ceria film connecting the tubes, the image contrast in the TEM experiment (Figure 7, left side) is comparable to that of the carbon-grid substrate surface, corresponding to only a few nanometres.

**Figure 8:**
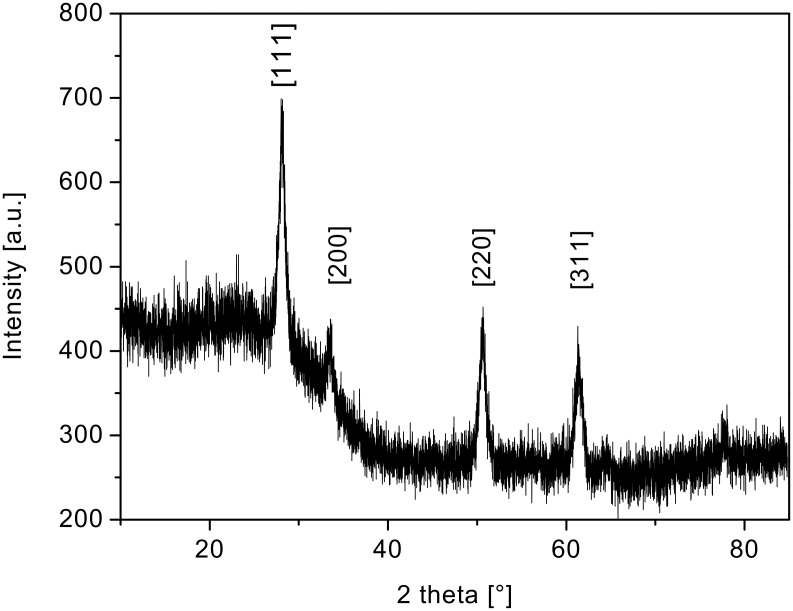
XRD spectra of nanostructured ceria (obtained with addition of Pluronic P123^®^ to the sol).

An average ceria particle size of about (5 ± 0.1) nm was deduced, by using the Scherrer equation, from the XRD measurements (Figure 8) of the ceria particles that constitute the film interconnecting the tubes. This is in good agreement with the ceria particle size obtained from the HRTEM studies but significantly larger than that observed for the ceria material obtained without Pluronic P123^®^ surfactant. Again the only phase observed in the XRD is the crystalline cubic-fluorite-type phase of CeO_2_.

**Figure 9:**
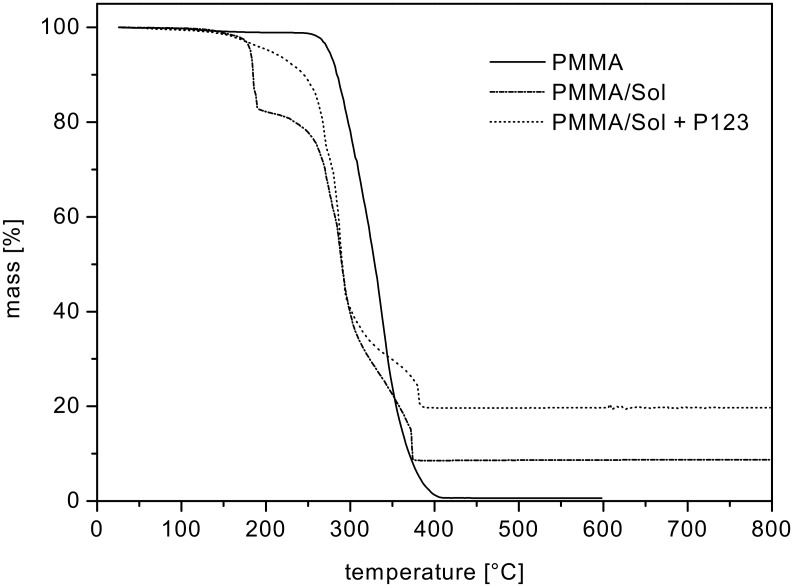
TGA measurements of bare PMMA fibres (PMMA, solid line), a PMMA/sol without Pluronic P123^®^ (PMMA/sol, dashed-dotted line) and a PMMA/sol containing Pluronic P123^®^ (PMMA/sol + P123, dotted line).

To study the thermal processing behaviour during the conversion process of the green body into the final ceria ceramics in more detail, we investigated the PMMA polymer fibres impregnated with ceria sol, with and without Pluronic P123^®^ surfactant (samples PMMA/sol and PMMA/sol + Pluronic P123^®^, Figure 9).

At 400 °C PMMA fibres were completely decomposed and removed from the samples. For the PMMA/sol with P123^®^ (PMMA/Sol + P123^®^) the transformation was already complete at 365 °C with a ceramic yield of nearly 20%. This finding is in contrast to the conversion of the PMMA/sol without additional P123^®^ surfactant (PMMA/sol) in which the overall ceramic yield was 10% and therefore significantly lower. This difference can be attributed to an enhanced wetting of the surface of the polymer fibres, as well as in the interstices between the packed PMMA fibre mats, during the impregnation step. This leads to a significantly enhanced wetting of the PMMA polymer template and thus a denser material deposition of the ceria sol.

**Figure 10:**
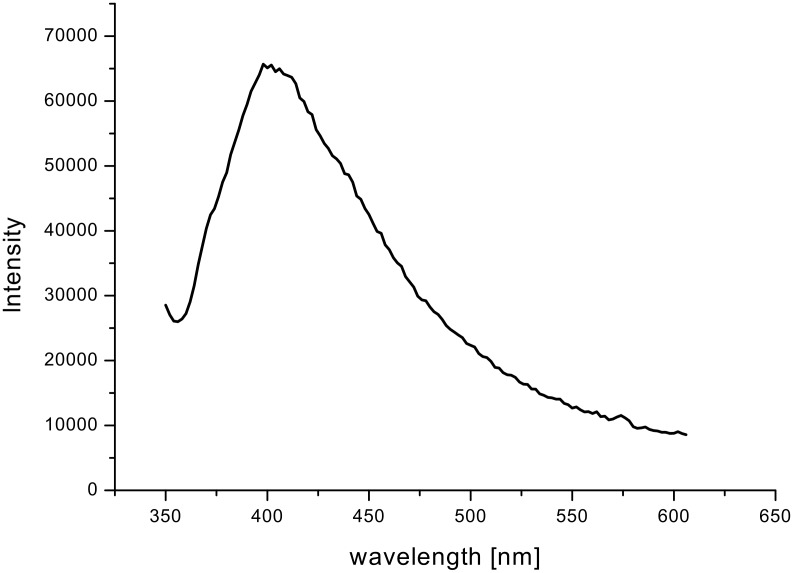
PL spectrum of microsized ceria tubes composed of nanostructured ceria (obtained with addition of Pluronic P123^®^ to the sol) at an excitation wavelength of 325 nm.

Photoluminescence (PL) measurements (Figure 10, excitation wavelength 325 nm) reveal a maximum at 415 nm [23]. The strong emission of CeO_2_ at this wavelength is related to abundant defects such as dislocations, which are helpful for fast oxygen transport [23].

#### Catalytic studies

Ceria nanomaterials as supports for precious metals (e.g., Au, Pt) show interesting properties in CO oxidation in the water gas shift reaction as well as in oxygen storage [12–14,24–25]. These properties are due to the high occurrence of oxygen defects in crystalline ceria. The relatively high surface area together with the nano/microsized morphology compared to nanocrystalline ceria renders our new ceria morphology interesting for catalysis. As a test reaction the synthesis of dimethyl carbonate (CH_3_O)_2_CO (DMC) by direct carboxylation of methanol was studied:


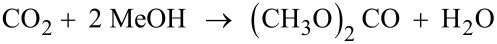


DMC is known as a green chemical and alternative to toxic and corrosive reagents, e.g., replacing phosgene or dimethyl sulfate (as a starting material for organic synthesis by carbonylation or methylation), as well as being an octane booster in gasoline and additive to diesel fuel (for particle emission decrease), and also a solvent [6,25–27]. Owing to its low toxicity, versatile reactivity and high dielectric constant, DMC also attracts interest as an electrolyte in lithium-ion batteries [28]. The formation of DMC by direct methanol carboxylation, however, is restricted by thermodynamic effects (equilibrium is far to the educt side) and, in addition to that, sometimes even by severe ceria catalyst agglomeration and decomposition [29]. The maximum yield of DMC observed is currently reported to be within 0.5 and 1 wt % [6,30–31]. CO_2_ as C_1_ feedstock is often used under supercritical conditions, affording an improved reactivity, polarity and solubility, thus enhancing its catalytic activity and selectivity [29,32]. Ceria shows both acidic and basic properties, which are important for its reactivity as a catalyst [29]. In addition, it is known that even though ceria and other electron deficient metal oxides, such as zirconia [30–31] and titania [33], are active in direct carboxylation of methanol to DMC, they are also easily deactivated, sometimes already before recycling experiments can be started, resulting in only marginal methanol conversion. It can be shown that this is due to ceria agglomeration and can be partially prevented by solid dilution of the active ceria catalyst with up to 20%, e.g., of alumina [34]. It would be interesting to find out whether a destructive agglomeration of the ceria catalyst could be diminished significantly by a nano/microsized ceria structure in which the nanoscaled particles are stabilized to a certain extent in a 1-D morphological order.

**Figure 11:**
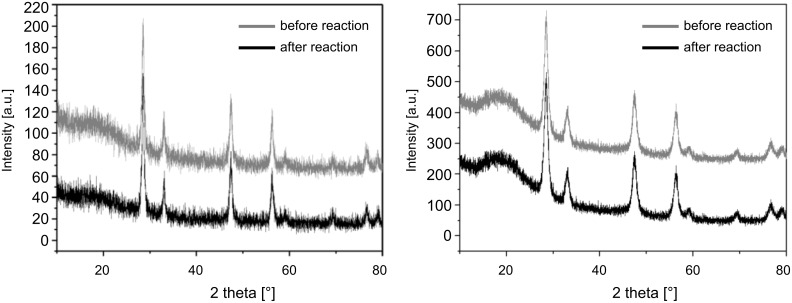
XRD spectra of the ceria reference sample (15 nm diameter) prepared by the standard oxalate-gel method (left) and sample 3 (right) obtained by template-directed synthesis with Pluronic P123^®^ before and after reaction, are shown.

The direct carboxylation reactions of methanol were conducted in a multibatch system (five parallel reactors) with independent pressure and temperature control for each reactor set up. Samples with different surface treatments were tested (ceria sample 1 was obtained by template-directed synthesis with Pluronic P123^®^ as surfactant, without additional plasma etching. Samples 2 and 3 were obtained as sample 1, but additional plasma etching was used with sample 3). Due to the plasma etching the tubular 1-D structure is more pronounced in sample 1 compared to 2 and 3. In all three samples the crystallite size is ca. 5 nm. So far, ceria with particle sizes between 15–60 nm have shown a maximum catalytic activity in the DMC synthesis reaction [30–31]. Therefore, a nanoparticulate reference sample of ceria with a crystallite size of 15 nm was prepared by the oxalate-gel precipitation technique [35]. Samples 1–3, as well as the ceria reference sample, maintained their structural integrity (ceria) before and after the catalytic reaction, as can be seen from the XRD spectra (see Figure 11 for sample 3, a similar behaviour was found for samples 1 and 2).

**Figure 12:**
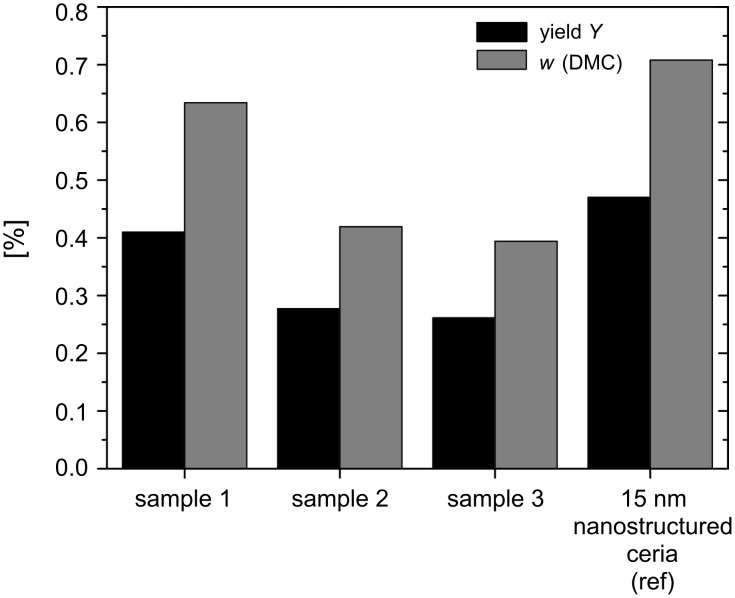
Comparison of the overall yield (*Y*) of nano/microsized ceria nanotubes (samples 1,2,3) versus nanostructured ceria particles (15 nm) as reference, and mass fractions of DMC in methanol (*w*) in the direct carboxylation reaction of methanol.

Sample 1 gave an overall mass fraction of 0.63 wt % DMC, which is 0.08 wt % less than for the 15 nm ceria reference sample (0.71 wt %). Samples 2 and 3 led to slightly lower yields of 0.42 wt % and 0.39 wt %, respectively, compared to the nanocrystalline ceria sample prepared by the oxalate-gel method. Related to methanol, the yields obtained from samples 1, 2 and 3 were 0.41, 0.28 and 0.26%, respectively, compared to 0.47 % from the 15 nm ceria reference sample (Figure 12, Table 1).

**Table 1:** Comparison of the experimental catalytic conditions in the direct carboxylation reaction of methanol for nano/microsized ceria nanotubes versus nanostructured ceria particles (15 nm diameter).^a^

catalyst sample	*m*_cat_ [mg]	*w*(DMC) [wt %]	*Y* [%]

1	104.2	0.63	0.41
2	96.1	0.42	0.28
3	99.9	0.39	0.26
ref	101.0	0.71	0.47

^a^*Y* = yield related to methanol; *w*(DMC) = mass fraction of DMC in MeOH; *m*_cat_ = catalyst mass (temperature = 150 °C, reaction time = 300 min; stirring rate = 600 rpm, pressure = 60 bar).

The 1-D ceria structures show slightly lower overall mass fractions of DMC and hence also lower yields than those of the ceria obtained by the oxalate-gel method. However, the catalyst activity is already close to the best values obtained from the reference sample. A crucial point relating to the morphology of the structure is their mechanical stability. We observed partial destruction of the 1-D structure during the catalytic reactions in the liquid reaction phase of the batch reactor setup. Currently we are setting up a gas-phase reaction system in order to study the DMC formation under gas phase conditions, with an aim to minimize the mechanical stress on the ceria-mat structure during the cycling experiments. This should avoid the mechanical breakdown of the nano/microsized 1-D structure into a nanoparticulate ceria material and could thus result in an even higher catalytic activity.

## Conclusion

Template-directed synthesis of ceria nanotubes was accomplished, using electrospun PMMA polymer templates. Exotemplating by spray coating of an inorganic ceria sol was used as the technique to introduce the ceria *after* final calcination. Templating under presence of Pluronic P123^®^ allows the formation of an interpenetrating network in which a thin ceria film interconnects the microsized ceria tubes. A combined process of plasma etching and calcination was chosen to remove the PMMA template material, avoiding thermal stress. The obtained highly crystalline 1-D ceria materials show a high activity in the direct carboxylation of methanol to dimethyl carbonate (DMC), but the mechanical stability of the 1-D material needs to be improved further.

## Experimental

### Synthesis of polymer template fibres

A solution of 15 wt % PMMA (type 7N, Röhm GmbH) in a mixture of acetone and dimethyl formamide (60/40; Merck KGaA) was electrospun from a glass syringe (5 mL) with tip dimensions of 0.8 × 40 mm at an electrode distance of 20 cm and 26 kV for 12 h. After 12 h, a dense web of PMMA fibres was collected on the copper counter electrode (15 × 15 cm^2^).

#### Preparation of ceria nanotubes

1.37 g (2.5 mmol) cerium ammonium nitrate (NH_4_)_2_Ce(NO_3_)_6_ (Alfa Aesar, 98.0+%) was dissolved in 40 mL distilled water. 0.65 mL aqueous 25 wt % ammonia solution (Merck) was added and the solution was vigorously stirred for half an hour. 1 g Pluronic P123^®^ (Sigma Aldrich) was added and dissolved at 50 °C. The sol was used immediately. The as-prepared cerium-containing sol was spray coated on the previously prepared polymer fibres by using a spray bottle (Roth, 50 mL). Afterwards, sol–gel transformation of the spray-coated sample was achieved in a furnace (Memmert) at 80 °C overnight. Plasma etching was performed by using a Diener Electronics PS Tech, Femto machine with 20 vol % O_2_ (Messer Griesheim) as etching gas, for 18 h. Finally, the green body obtained was calcined at 350 °C for 3 h.

#### Preparation of ceria reference sample

Reference samples (ref) of ceria were prepared by oxalate-gel precipitation [35]. A freshly prepared solution of 1 mol·L^−1^ oxalic acid (≥99.0%, Sigma-Aldrich) in ethanol (≥99.8%, 1% methyl ethyl ketone, Carl Roth) was added under vigorous stirring to an ethanolic solution of cerium(III) nitrate hexahydrate (≥99.5%, p.a, Carl Roth, 0.33 mol·L^−1^) with 20% molar excess. A white gel formed instantaneously and was subsequently aged for an additional two hours at room temperature under medium stirring. Afterwards the aged gel was heated at 80 °C on a heating blanket to dryness and calcined in air (25 mL·g^−1^·min^−1^) at 600 °C (RT, 1 h to 150 °C, 4.5 h to 600 °C, 4 h at 600 °C).

#### Direct carboxylation of methanol

For the catalytic reaction 10 mL methanol (250 mmol, ≥99.9%, Carl Roth, 0.02 wt % water) and 100 mg catalyst were charged into the reactor. After closure the reactors were rendered inert by flushing three times with argon (5.0, Linde) subsequently followed by pressurizing the reactor with 55 bar CO_2_ (4.5, Linde) for one minute. The reactors were heated up to 150 °C and the temperature was held for the duration of the reaction. After reaction, the reactors were quenched to room temperature and the pressure was released slowly. Samples were taken and were characterized quantitatively by GC analysis with toluene as internal standard.

#### Materials characterization

Scanning electron microscopy (SEM) was performed on a FEI XL30 FEG at an operating voltage of 25 kV. For transmission electron microscopy (TEM) a FEI Technai F20 was used, with an operating voltage of 200 keV. X-ray diffraction (XRD) spectra were recorded on StoeCIE and StadiP (Co Kα1) instruments. Thermogravimetric analysis (TGA) was performed on a Netzsch TG 209 F1 instrument coupled with a QMS 403 C mass spectrometer. For BET measurements a NOVA 3000e (Quantachrome) was used. For PLE measurements, Horiba, Fluorog-3 (Xe, excitation wavelength 325 nm) was used. For UV–vis spectroscopy, Perkin Elmer, Lambda 900 was used. DLS was measured by using a Malvern Zetasizer Nano with a red laser of 633 nm.
